# A photon-controlled diode with a new signal-processing behavior

**DOI:** 10.1093/nsr/nwac088

**Published:** 2022-05-10

**Authors:** Shun Feng, Ruyue Han, Lili Zhang, Chi Liu, Bo Li, Honglei Zhu, Qianbing Zhu, Wei Chen, Hui-Ming Cheng, Dong-Ming Sun

**Affiliations:** Shenyang National Laboratory for Materials Science, Institute of Metal Research, Chinese Academy of Sciences, Shenyang 110016, China; School of Physical Science and Technology, ShanghaiTech University, Shanghai 200031, China; Shenyang National Laboratory for Materials Science, Institute of Metal Research, Chinese Academy of Sciences, Shenyang 110016, China; School of Materials Science and Engineering, University of Science and Technology of China, Shenyang 110016, China; Shenyang National Laboratory for Materials Science, Institute of Metal Research, Chinese Academy of Sciences, Shenyang 110016, China; Shenyang National Laboratory for Materials Science, Institute of Metal Research, Chinese Academy of Sciences, Shenyang 110016, China; School of Materials Science and Engineering, University of Science and Technology of China, Shenyang 110016, China; Shenyang National Laboratory for Materials Science, Institute of Metal Research, Chinese Academy of Sciences, Shenyang 110016, China; School of Materials Science and Engineering, University of Science and Technology of China, Shenyang 110016, China; Shenyang National Laboratory for Materials Science, Institute of Metal Research, Chinese Academy of Sciences, Shenyang 110016, China; School of Materials Science and Engineering, University of Science and Technology of China, Shenyang 110016, China; Shenyang National Laboratory for Materials Science, Institute of Metal Research, Chinese Academy of Sciences, Shenyang 110016, China; School of Materials Science and Engineering, University of Science and Technology of China, Shenyang 110016, China; Shenyang National Laboratory for Materials Science, Institute of Metal Research, Chinese Academy of Sciences, Shenyang 110016, China; School of Materials Science and Engineering, University of Science and Technology of China, Shenyang 110016, China; Shenyang National Laboratory for Materials Science, Institute of Metal Research, Chinese Academy of Sciences, Shenyang 110016, China; School of Materials Science and Engineering, University of Science and Technology of China, Shenyang 110016, China; Faculty of Materials Science and Engineering/Institute of Technology for Carbon Neutrality, Shenzhen Institute of Advanced Technology, Chinese Academy of Sciences, Shenzhen 518055, China; Shenyang National Laboratory for Materials Science, Institute of Metal Research, Chinese Academy of Sciences, Shenyang 110016, China; School of Materials Science and Engineering, University of Science and Technology of China, Shenyang 110016, China

**Keywords:** photon-controlled diode, fully-off, rectifying, photomemory array

## Abstract

The photodetector is a key component in optoelectronic integrated circuits. Although there are various device structures and mechanisms, the output current changes either from rectified to fully-on or from fully-off to fully-on after illumination. A device that changes the output current from fully-off to rectified should be possible. We report the first photon-controlled diode based on a n/n^−^ molybdenum disulfide junction. Schottky junctions formed at the cathode and anode either prevent or allow the device to be rectifying, so that the output current of the device changes from fully-off to rectified. By increasing the thickness of the photogating layer, the behavior of the device changes from a photodetector to a multifunctional photomemory with the highest non-volatile responsivity of 4.8 × 10^7^ A/W and the longest retention time of 6.5 × 10^6^ s reported so far. Furthermore, a 3 × 3 photomemory array without selectors shows no crosstalk between adjacent devices and has optical signal-processing functions including wavelength and power-density selectivity.

## Teaser

Photon-controlled diodes, as a new circuit element proposed for the first time, change the output current from a fully-off state to a rectified state after illumination, which paves the way for future highly integrated, low-power, intelligent optoelectronic systems.

## INTRODUCTION

Transistor and integrated circuit (IC) technology has achieved tremendous developments over the past 70 years. As the size of device components approaches the technical and physical limits, ICs will, on the one hand, see sizes decrease and 3D integration [1] and, on the other, see more diversified uses including neuromorphic sensing and computing chips [2], photonic integrated chips [3,4] and quantum computing chips [5]. Among them, photonic integrated chips have light emission, modulation, transmission and detection abilities, which can integrate optical transmission and information processing, thereby supporting chip development for large capacity, low power consumption, large-scale integration and artificial intelligence [3,4].

**Figure 1.**
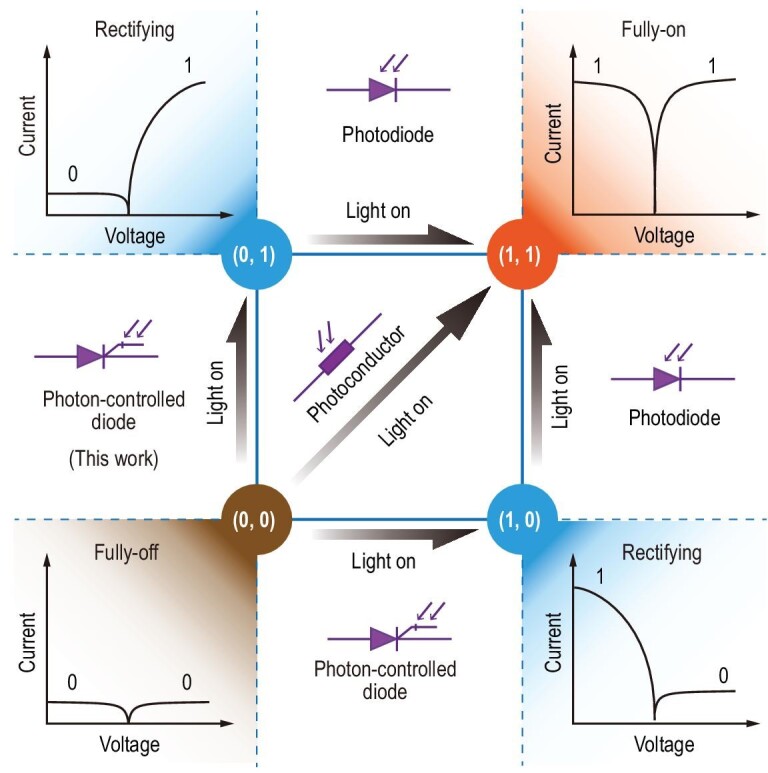
The three fundamental current states of photodetectors: fully-off (0, 0), fully-on (1, 1) and rectifying (0, 1) or (1, 0). For a photodiode, the output current changes from rectified to fully-on; for a photoconductor or phototransistor, the output current changes from fully-off to fully-on after illumination. For the photon-controlled diode as a ‘missing’ element, the output current will change from fully-off to rectified.

A photodetector is an important semiconductor device that can detect optical signals and convert them into electrical signals. Typical devices include photodiodes, phototransistors and photoconductors [6,7]. Although there are many types of photodetectors with different mechanisms and structures, their representative behavior can be summarized as a limited number of actions depending on their different electrical output characteristics after illumination. Figure 1 shows the output current–voltage relationships of a photodetector before and after being excited by light. The three typical states are fully-off (0, 0), fully-on (1, 1) and rectifying (0, 1) or (1, 0). For example, the output current of a photodiode changes from rectified to fully-on after illumination, whereas the output current of a photoconductor or a phototransistor changes from fully-off to fully-on.

From the perspective of the signal-change behavior shown in Fig. 1, there should be a new device that changes the output current from fully-off to rectified. As a ‘missing’ element, such a device has not yet been discovered and will play a key role in future optoelectronic systems, such as optical logic [8–10], high-precision imaging [11–15] and information processing [16–35]. For instance, we can use optical signals to control the logic functions of optoelectronic devices, greatly improve the ability and effectiveness of light control and reform the existing photoelectric conversion structure and fundamental logic cognition. In addition, rectification controlled by light can avoid the crosstalk issue of photodetector arrays without selectors, thereby helping to further improve the integration of the array.

We believe that this is the first report of a photon-controlled diode based on a n/n^−^ molybdenum disulfide (MoS_2_) junction. Controlled by light, the Schottky junctions formed at the cathode and anode suppress or show the rectification property of the n/n^−^ junction, so that the output current of the device changes from fully-off to rectified. As a photodetector, its responsivity exceeds 10^5^ A/W. By increasing the thickness of the photogating layer, the behavior of the device changes from being a photodetector to a multifunctional photomemory with the highest non-volatile responsivity of 4.8 × 10^7^ A/W and the longest retention time of 6.5 × 10^6^ s reported so far. We have also fabricated a 3 × 3 photomemory array without any selectors, showing no crosstalk, as well as optical signal detection and processing functions.

## RESULTS AND DISCUSSION

**Figure 2.**
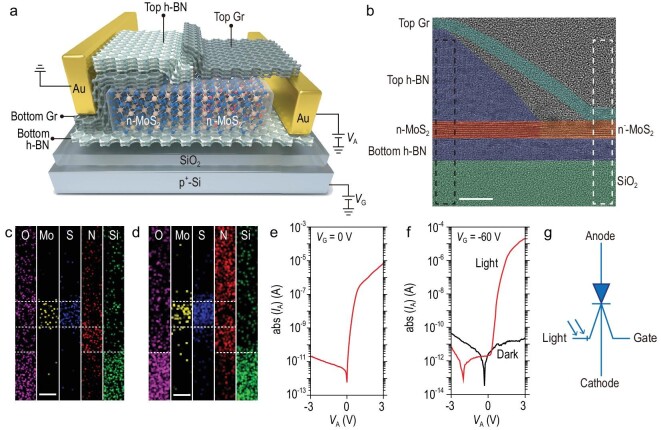
Device structure and characterization. (a) Schematic of a photon-controlled diode fabricated by sandwiching a h-BN layer between a n/n^−^ MoS_2_ junction and a SiO_2_/p^+^-Si back-gate, using bottom/top graphene as the cathode/anode and a top h-BN as the protecting mask. (b) False-color TEM image of the cross section showing the high-quality interfaces of the heterostructures (scale bar: 10 nm). (c) Elemental maps of O, Mo, S, N and Si in the white dashed rectangular region in Fig. 2b, where O can be detected in the n^−^-MoS_2_ region (scale bar: 5 nm). (d) Elemental maps of O, Mo, S, N and Si in the black dashed rectangular region in Fig. 2b, where O is not detected in the n-MoS_2_ region (scale bar: 5 nm). (e) abs (*I*_A_)–*V*_A_ characteristics of the photon-controlled diode in the dark at *V*_G_ = 0 V. (f) abs (*I*_A_)–*V*_A_ characteristics at *V*_G_ = –60 V in the dark and under 405 nm of light illumination with a power density of 32 μW/cm^2^. (g) A symbol for the photon-controlled diode as a circuit element.

The photon-controlled diode consists of a lateral n/n^−^ MoS_2_ junction, bottom and top graphene (Gr) as cathode and anode, and a SiO_2_/p^+^-Si back-gate stack. The lightly p-doped MoS_2_ (n^−^-MoS_2_) was obtained from the as-transferred MoS_2_ (n-MoS_2_) using an oxygen plasma treatment [36–38] in which a top hexagonal boron nitride (h-BN) layer was used as a protecting mask for the n-MoS_2_ underneath and a bottom h-BN layer was sandwiched between the n/n^−^ MoS_2_ junction and the SiO_2_/p^+^-Si back-gate (Fig. 2a; Supplementary Figs 1 and 2; ‘Methods’). The cross section of the device shows a van der Waals heterojunction of MoS_2_ and h-BN without any gaps, obvious defects and contamination (Fig. 2b). Although oxygen plasma treatment destroyed the lattice on the surface of the MoS_2_ materials, a lateral n/n^−^ MoS_2_ junction was still formed inside the materials. Oxygen is detected in n^−^-MoS_2_ by energy-dispersive X-ray spectroscopy (EDX) and this reduces the electron concentration in the as-transferred n-MoS_2_ (Fig. 2c; Supplementary Figs 3 and 4). On the other hand, oxygen is not detected in n-MoS_2_, confirming its intact contact with the top h-BN layer (Fig. 2d).

When the applied gate voltage (*V*_G_) is 0 V, the current–voltage (*I*_A_–*V*_A_) characteristics of the photon-controlled diode show a rectifying behavior, with an on/off current ratio of >10^5^ and a low off current of ∼10^–10^ A at *V*_A_ = −3 V (Fig. 2e). In contrast, the current of the Gr/n-MoS_2_/Gr and Gr/n^−^-MoS_2_/Gr structures at *V*_A_ = ±3 V is >10^–6^^ ^A, which indicates that the rectifying behavior of the photon-controlled diode comes from the n/n^−^ MoS_2_ junction (Supplementary Fig. 4). The photon-controlled diode can work in a gate voltage-controlled mode, i.e. the output current changes from a fully-off state to a rectifying state when *V*_G_ is changed from −60 to 60 V (Supplementary Fig. 5). It can also work in a photon-controlled mode, i.e. when *V*_G_ = −60 V, it is in the fully-off state in the dark but illumination with 405 nm of light produces a rectifying state with an on/off current ratio of >10^6^ (Fig. 2f). Therefore, by changing the light illumination and using a constant bias gate, a new signal-processing behavior of changing from fully-off to rectifying is realized (Fig. 2g).

**Figure 3.**
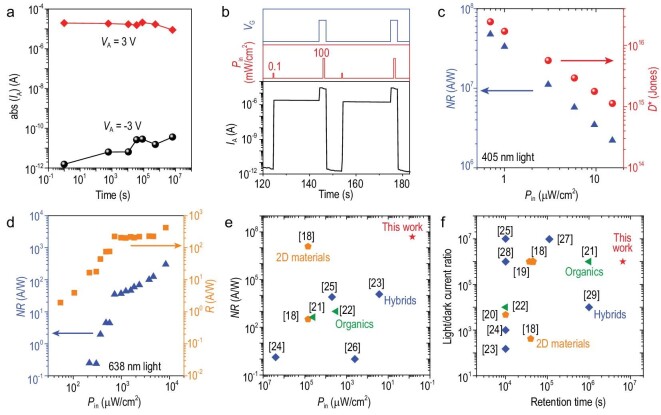
Photomemory characteristics of the photon-controlled diode. (a) Current retention property. *V*_A _=_ _±3 V, *V*_G _=_ _–60 V. (b) Switching characteristics. For the programming process, 405 nm of light with a power density (*P*_in_) of 0.1 mW/cm^2^ was applied for 0.5 s at a negative *V*_G_, whereas in the erasing process, 405 nm of light with *P*_in_ of 100 mW/cm^2^ was applied for 1 s at a positive *V*_G_. The overshoot of *I*_A_ in the erasing process is because of the changing of *V*_G_. (c) Non-volatile responsivity (*NR*) and detectivity (D^*^) as a function of *P*_in_ using 405 nm of light. *V*_A_ = 3 V, *V*_G_ = –60 V. *NR* = (*I*_store_ – *I*_dark_)/*P*_in_, where *I*_dark_ is the dark current and *I*_store_ is the storage current. D^*^ = (*AB*)^1/2^*NR*/*S*^1/2^, where *A* is the active area of 30 μm^2^, *B* is the bandwidth of 1 Hz and *S* is the noise power spectral density. (d) Responsivity (*R*) and *NR* as a function of *P*_in_ using 638 nm of light. *R* = (*I*_ph_ – *I*_dark_)/*P*_in_, where *I*_ph_ is the photocurrent. *V*_A_ = 3 V, *V*_G_ = –60 V. (e and f) Benchmarks of the photomemory characteristics of the photon-controlled diode showing it has the highest reported *NR* and the longest reported retention time.

As a photodetector, the responsivity of this device is >10^5^ A/W with a response time of <1 s (Supplementary Fig. 6). By increasing the thickness of the bottom h-BN layer from one to several nanometers, the behavior of the photon-controlled diode is changed from an ordinary photodetector to a new type of photomemory. The retention characteristics of the device show that the on/off current ratio is >10^6^ after illumination and remains at >10^5^ for 6.5 × 10^6^ s (Fig. 3a; Supplementary Fig. 7). By extrapolation of the retention current, the stored data can be securely extracted with an on/off current ratio at *V*_A_ = ±3 V of >10^3^ for ≤10^9^ s (Supplementary Fig. 7). The switching characteristics of the device show that the light/dark current ratio is >10^6^ (Fig. 3b) and the device has a stable multi-level storage ability (Supplementary Fig. 8).

The photon-controlled diode has a wavelength-dependent responsivity. It is sensitive to 405 nm of light with a non-volatile responsivity (*NR*) of 4.8 × 10^7^ A/W and a detectivity (D^*^) of 2.4 × 10^16^ Jones at a light power density (*P*_in_) of 0.7 μW/cm^2^ (Fig. 3c; Supplementary Fig. 9). In contrast, it is much less sensitive to 638 nm of light, showing a responsivity (*R*) of <10^3^ A/W and a relatively lower *NR* (Fig. 3d; Supplementary Fig. 10).

In order to benchmark the photomemory characteristics of our photon-controlled diode, its performance was compared with those of devices composed of various 2D [18–20], organic [21,22] and hybrid [23–28] materials (Fig. 3e and f). Our device shows the highest *NR* and the longest retention time (Supplementary Table 1). It is worth noting that photon-controlled diodes using other 2D materials (such as WS_2_) can be fabricated using similar methods, which provides many design possibilities for the expected signal-processing behaviors (‘Methods’; Supplementary Fig. 11).

**Figure 4.**
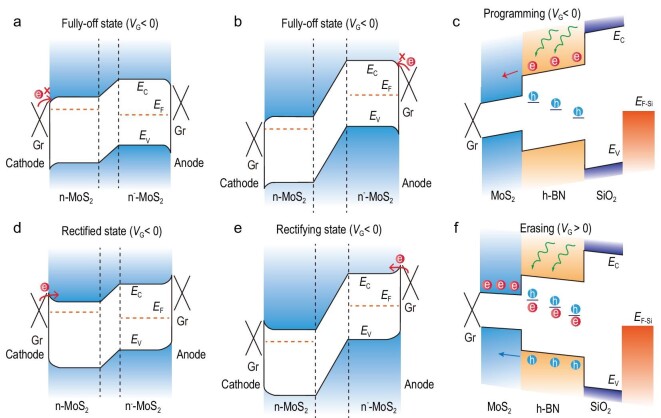
Energy-band diagrams illustrating photomemory mechanism. (a) The fully-off state (*V*_A_ > 0). (b) The fully-off state (*V*_A_ < 0). (c) During the programming process. (d) In the rectifying state (*V*_A_ > 0). (e) In the rectifying state (*V*_A_ < 0). (f) During the erasing process. *E*_C_ and *E*_V_ are respectively the conduction-band minimum and the valance-band maximum of MoS_2_. *E*_F_ and *E*_F-Si_ are the Fermi energy levels of MoS_2_ and p^+^-Si, respectively. *e* and *h* are respectively electrons and holes.

The photon-controlled diode is essentially a n/n^−^ MoS_2_ junction inserted between two Gr/MoS_2_ Schottky junctions at the cathode and the anode. By controlling the light, the Schottky junction suppresses or permits the rectification behavior of the n/n^−^ junction, so that the output current of the photon-controlled diode changes from fully-off to rectified. Figure 4a and b shows the energy-band diagram in the fully-off state. When a negative *V*_G_ is applied in the dark, the electron potential barriers at the cathode and anode increase, so that electron conduction is not possible, leading to the fully-off state.

Figure 4c shows the energy-band diagram of the device during the programming process. When it is exposed to 405 nm of light, electrons are excited by the photons from the defect energy levels of the bottom h-BN layer to the conduction band [18,39] and then move to the MoS_2_ conduction band at a negative *V*_G_. The bottom h-BN layer acts as a photogating layer in which the remaining holes at the defect energy levels offset the effect of the negative *V*_G_. Therefore, the electron potential barriers at the cathode and the anode are reduced and electrons can pass through the Gr/MoS_2_ Schottky junctions because of the tunneling effect, showing the rectification behavior of the n/n^−^ MoS_2_ junction (Fig. 4d and e). When 638 nm of light is used, the photon energy is lower, which mainly excites carriers in MoS_2_ to produce a photo-conduction effect, leading to a relatively lower *R*. When the light is off, photo-generated carriers in MoS_2_ recombine with each other. Meanwhile, only electrons in shallow levels in h-BN are excited to its conduction band and these are very limited, leading to a low *NR.*

When a thin h-BN was used for the photogating layer, the device worked as a photodetector. Because of the relatively thin tunneling barrier, the excited electrons can move back from the MoS_2_ to the defect energy levels of h-BN after removal of light and *V*_G_ (Supplementary Fig. 6). However, when a thick h-BN layer of about several nanometers was used for the photogating layer, few electrons can return to the defect energy levels of h-BN after removing the light and *V*_G_ due to the thick tunneling barrier, so the photogating effect of the h-BN remains and the photon-controlled diode works as a photomemory. Figure 4d shows the energy-band diagram of the device during the erasing process. When a positive *V*_G_ and 405 nm of light are used, electrons are excited from the valance band to the defect energy levels of h-BN and recombine with holes [18,39] (Fig. 4f).

**Figure 5.**
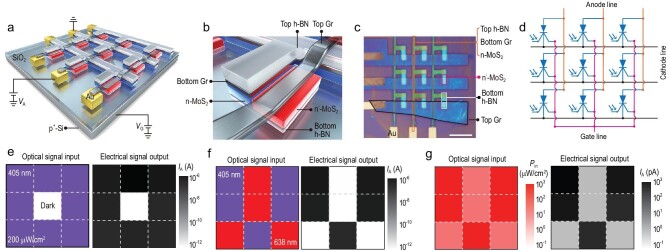
A photomemory array. (a) Schematic of a 3 × 3 photomemory array without selectors using a MoS_2_ photon-controlled diode as a unit. (b) Magnified image of the individual unit. (c) Optical photograph of the fabricated array (scale bar: 10 μm). (d) Equivalent circuit. (e) Lack of crosstalk in the photomemory array without selectors. (f) Demonstration of the wavelength selectivity of 405 nm (purple region) and 638 nm (red region) of light with the same power density of 200 μW/cm^2^ in the array. (g) Demonstration of the power-density selectivity for the same 638 nm light. The red and pink regions in the array represent different power densities of 780 and 26 μW/cm^2^, respectively.

A 3 × 3 photomemory array without selectors was designed with the MoS_2_ photon-controlled diodes as pixel units (Fig. 5; Supplementary Fig. 12; ‘Methods’). All nine devices in the array worked well and had a similar performance, indicating a good device uniformity (Supplementary Fig. 13). Figure 5e shows no crosstalk in the photomemory array. When the optical signal input is provided to all pixel units except the central one, the electrical signal output exhibits a light/dark current ratio of >10^5^ even if none of the external selectors is used. During the measurement of any individual device in the array, all possible sneak paths are open. Since there is at least one reverse-biased diode in a sneak path, the effects of crosstalk are avoided.

The lack of crosstalk in the photomemory array also enables optical signal-processing functions such as wavelength selectivity and power-density selectivity. An optical signal input composed of both 405 and 638 nm wavelengths of light was used to demonstrate the wavelength selectivity and the electrical signal output showed a clear pattern with a light/dark current ratio of >10^5^ (Fig. 5f). Different power densities of 780 and 26 μW/cm^2^ of the 638 nm of light were used to demonstrate the power-density selectivity and the electrical signal output showed a pattern with a light/dark current ratio of >230 (Fig. 5g). The wavelength and power-density-dependent responsivity of the photon-controlled diode can be used to reduce noise signals and achieve a high contrast and high-resolution imaging. It is also important in applications such as optical information demodulators [26] and neuromorphic vision systems [32–34].

## CONCLUSION

Using a n/n^−^ MoS_2_ junction, we have designed and fabricated a photon-controlled diode with an unusual signal-processing behavior that can change the output current from fully-off to rectified after illumination. When a thinner photogating layer was used, the device worked as a photodetector with a responsivity of >10^5^ A/W, whereas when a thicker photogating layer was used it worked as a photomemory with the highest *NR* (4.8 × 10^7^ A/W) and the longest retention time (6.5 × 10^6^ s) reported so far. Furthermore, a 3 × 3 photomemory array without any selectors showed no crosstalk as well as wavelength and power-density selectivity. The proposed photon-controlled diode is the first to demonstrate this new signal-processing behavior. It is a new circuit element that has been a ‘missing element’ and it should pave the way for future high-integration, low-power and intelligent optoelectronic systems.

## METHODS

### Device fabrication

Step 1: material preparation. Graphene, MoS_2_, WS_2_ and h-BN were exfoliated from their bulk crystals using Scotch® tape and were placed on a SiO_2_/p^+^Si substrate. Step 2: top h-BN layer patterning. A polymethyl methacrylate (PMMA) layer (495K MW, A4, MicroChem) was spin-coated on the h-BN/SiO_2_/p^+^Si substrate at 2000 rpm and baked at 190°C for 5 min, and then another PMMA layer (950K MW, A2, MicroChem) was spin-coated at 4000 rpm and baked at 190°C for 2 min. An undercut structure was created using electron-beam lithography (EBL) and a developing process. Subsequently, the h-BN flakes were patterned using reactive ion etching (RIE) (CHF_3_ with a flux rate of 20 sccm; O_2_ with a flux rate of 4 sccm; pressure, 2.0 Pa; power, 50 W; etching time, 1 min) and lift-off processes. Step 3: heterostructure stacking. The patterned top h-BN layer was picked up using a piece of propylene-carbonate and the bottom graphene layer (used as the cathode) and n-MoS_2_ (or n-WS_2_) layer were then picked up in sequence. The stack was released onto a bottom h-BN photogating layer on a SiO_2_/p^+^Si substrate at 130°C, followed by heating at 350°C for 120 min in vacuum to remove the propylene-carbonate. Step 4: metal-contact deposition. Metal contacts (Ti/Au: 5/50 nm) were formed using EBL, RIE (CHF_3_ with a flux rate of 20 sccm; O_2_ with a flux rate of 4 sccm; pressure, 2.0 Pa; power, 50 W; etching time, 1 min), electron-beam evaporation and lift-off processes. Step 5: n/n^−^ MoS_2_ junction formation. The n^−^-MoS_2_ was formed using an oxygen plasma treatment (O_2_ with a flux rate of 180 sccm; power, 200 W; time, 60 min) on the as-transferred n-MoS_2_. The patterned top h-BN serves as a protecting mask layer for the n-MoS_2_ underneath. Step 6: anode formation. Polydimethylsiloxane was used as the medium to transfer the top graphene layer onto the n^−^-MoS_2_ to form the anode.

### Characterization

The materials and devices were characterized using an optical microscope (Nikon ECLIPSE LV100ND), aberration-corrected TEM (Thermo Scientific^TM^, Titan Cube Themis G2), with the operating voltage at 300 kV and Super-X detector system for Energy-Dispersive X-ray spectrometry (EDX) mappings, and an X-ray photoelectron spectroscopy analyser (Thermo VG Scientific ESCALAB250). The electrical and optoelectronic performance was measured using a semiconductor analyser (Agilent B1500A), a probe station (Cascade M150) and a laser diode controller (Thorlabs ITC4001, with laser excitations of 405 and 638 nm) in a dark room at room temperature; 405 nm of light was generated using a Thorlabs ITC4001 unit, and a current amplifier (Model SR570) and an oscilloscope (Tektronix MDO3102) were used to provide a gate voltage to characterize the programming and erasing performance. The noise was measured using a noise-measurement system (Fs Pro, 100 kHz bandwidth).

## Supplementary Material

nwac088_Supplemental_File
