# Supplementary material for: A photon-controlled diode with a new signal-processing behavior
Source: Natl Sci Rev. 2022 May 10;9(8):nwac088. doi: 10.1093/nsr/nwac088 (PMC9385463; doi:10.1093/nsr/nwac088)
Supplement: nwac088_Supplemental_File [file nwac088_supplemental_file.docx]

**Supplementary Information**

**A photon-controlled diode with a new signal processing behavior**

Feng *et al*.


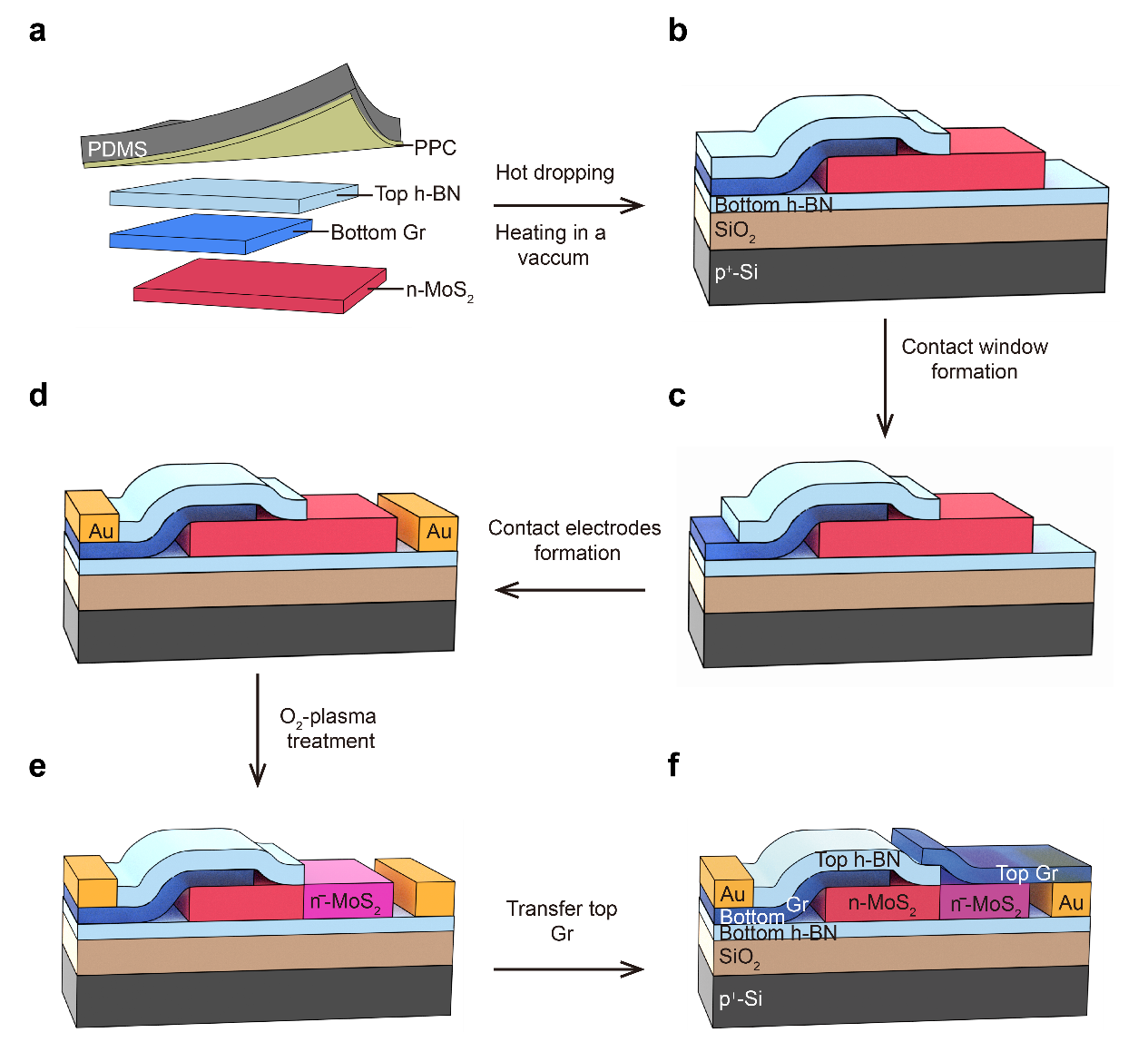


**Supplementary Fig. 1** Illustration of the device fabrication. (a) h-BN etching mask, bottom graphene (Gr) and MoS_2_ layers were picked up by a polydimethylsiloxane (PDMS)/propylene-carbonate (PPC) layer. (b) The structure was stacked on a h-BN photogating layer at 130°C, followed by heating in a vacuum at 350°C for 120 min to remove the PPC. (c) A contact window was formed by electron-beam lithography (EBL), reactive ion etching (RIE) and lift-off processes. (d) Metal contacts (Ti/Au: 5/50 nm) were formed by EBL, RIE, electron-beam evaporation and lift-off processes. (e) p-type doping of MoS_2_ by oxygen (O_2_) plasma treatment. (f) Transferring the top graphene electrode.


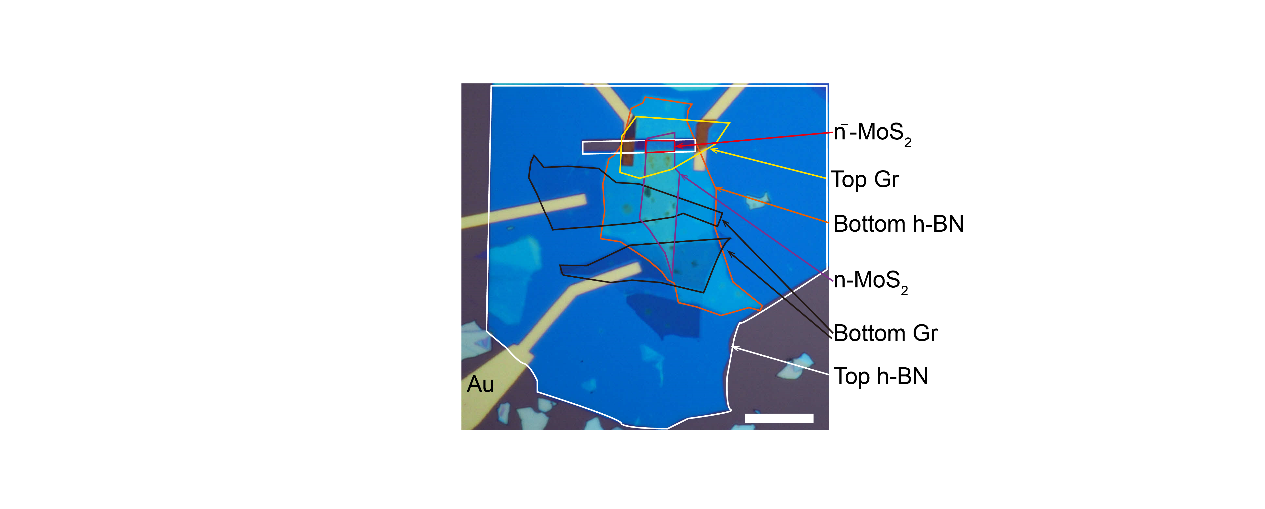


**Supplementary Fig. 2** Optical image of the photon-controlled diode (scale bar: 10 μm). The different materials are outlined and labelled.


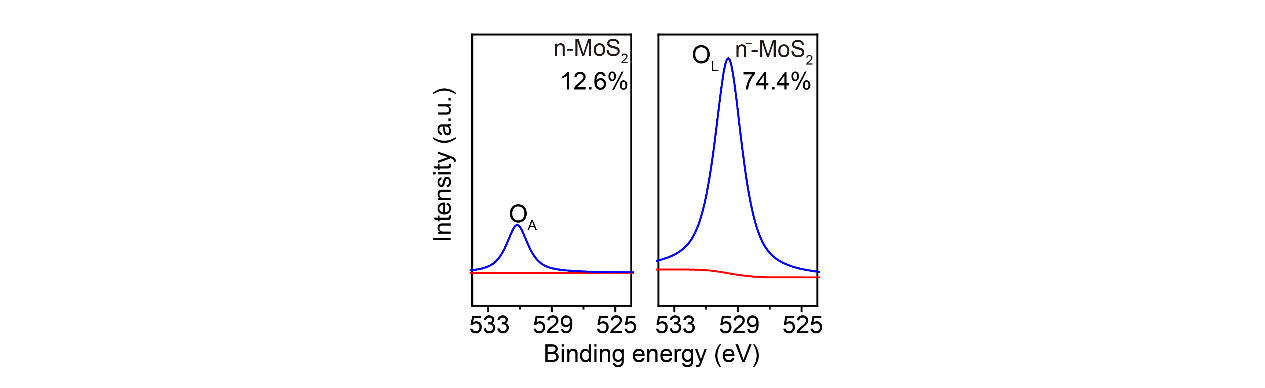


**Supplementary Fig. 3** X-ray photoelectron spectroscopy (XPS) of pure MoS_2_ (n-MoS_2_) flakes and O_2_ plasma treated MoS_2_ (n^−^-MoS_2_) flakes for O 1s. O_A_ is the adsorbed oxygen and O_L_ is the lattice oxygen. After the O_2_ plasma treatment the MoS_2_ crystal was doped with oxygen.


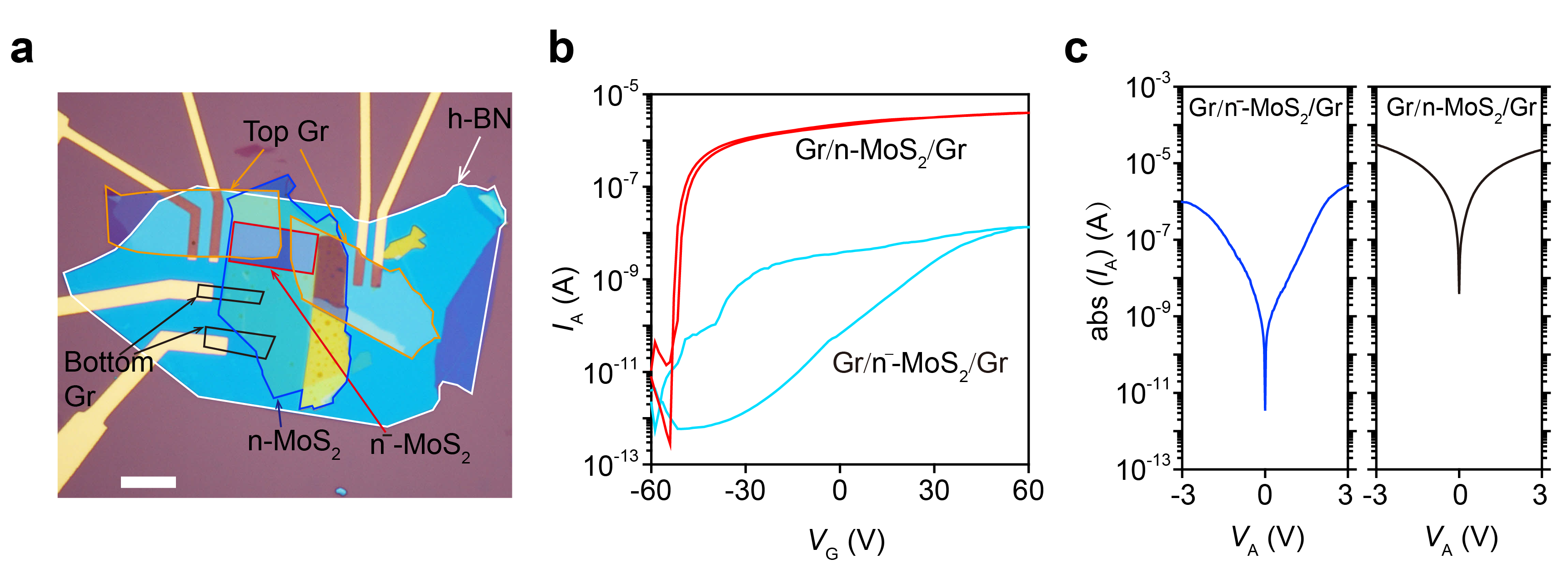


**Supplementary Fig. 4** (a) Optical image of a n/n^−^ MoS_2_ junction, two graphene electrodes are used for each of n-MoS_2_ and n^−^-MoS_2_, (scale bar: 5 μm). (b) *I*_A_ -*V*_G_ characteristics for Gr/n-MoS_2_/Gr and Gr/n^−^-MoS_2_/Gr junctions at *V*_A_ of 0.1 V, indicating effective p-doping of n^−^-MoS_2_. (c) abs (*I*_A_) -*V*_A_ characteristics of the Gr/n^−^-MoS_2_/Gr and Gr/n-MoS_2_/Gr devices at *V*_G_ = 0 V.


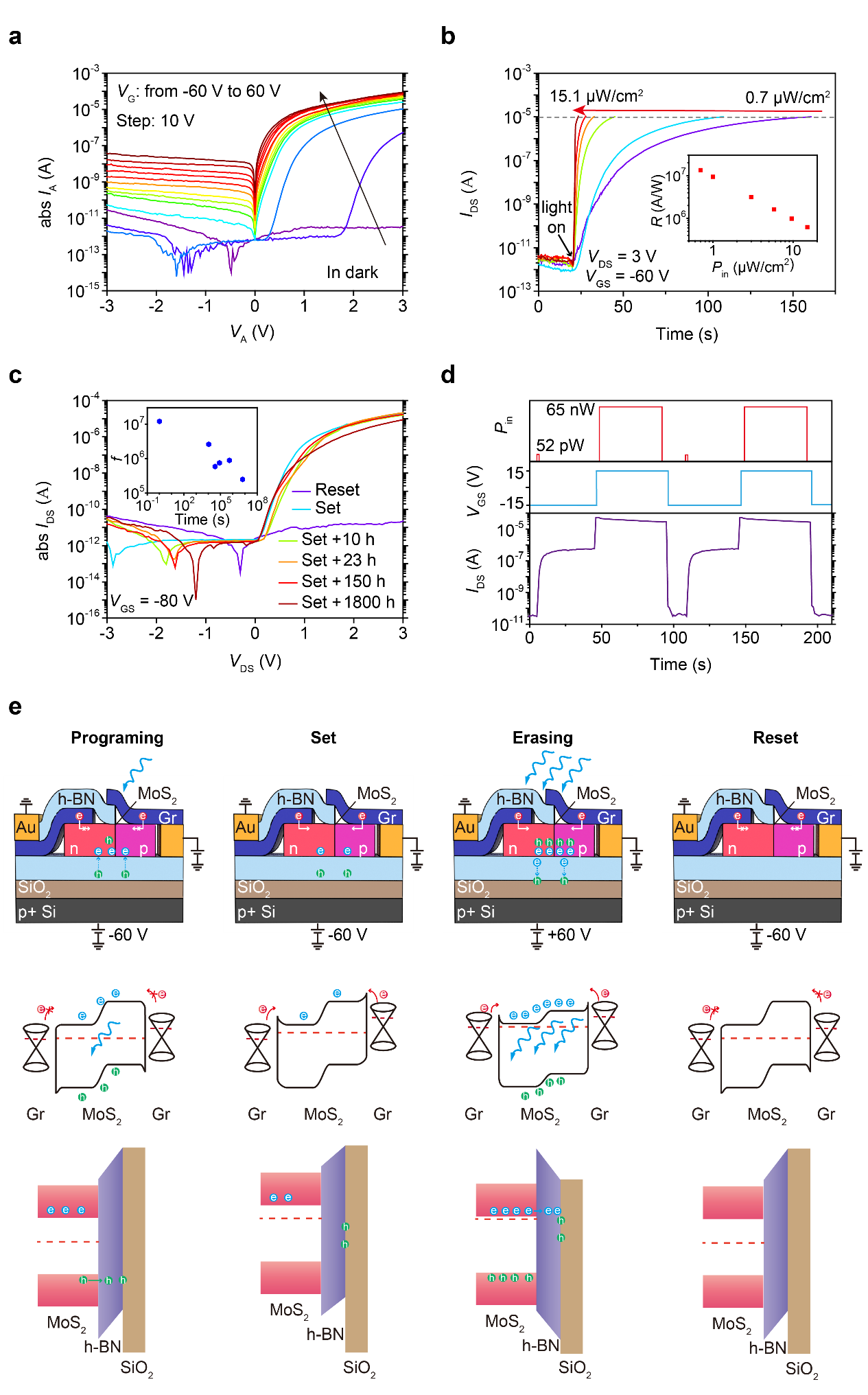


**Supplementary Fig. 5** abs (*I*_A_)-*V*_A_ characteristics of the device in the dark with *V*_G_ changing from −60 V to 60 V in 10 V steps. As the gate voltage increases, rectification emerges.


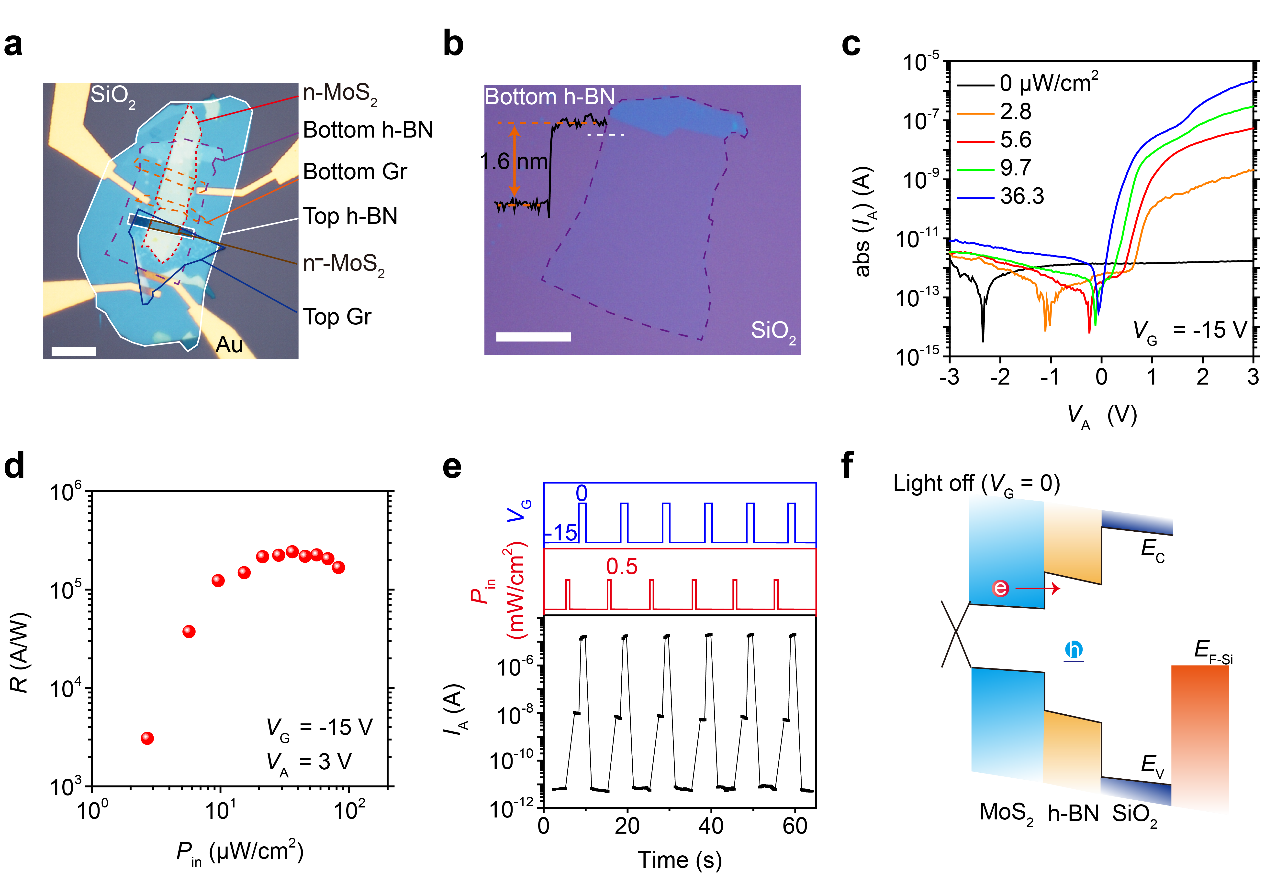


**Supplementary Fig. 6** Characteristics of a photodetector based on a MoS_2_ photon-controlled diode. (a) Optical image of the device (scale bar: 10 μm). The outlines denote the areas of the different materials. The thickness of SiO_2_ layer is 100 nm. (b) Optical image of the bottom h-BN (scale bar: 10 μm). Inset: Height profile along the white dashed line. (c) abs (*I*_A_)-*V*_A_ characteristics of the device under 405 nm light at *V*_G_ = −15 V. (d) Responsivity (*R*) as a function of *P*_in_ under 405 nm light at *V*_A_ = 3 V and *V*_G_ = –15 V. (e) Switching characteristics of the device under 405 nm light with a power density of 0.5 mW/cm^2^ (light pulse width 1 s) at *V*_A_ = 3 V and *V*_G_ = –15 V and a reset voltage pulse (–15 to 0 V, pulse width 2 s) to the gate electrode. (f) Mechanism of the photon-controlled diode as a photodetector. Energy band diagrams for the cases of light off. *E*_C_, *E*_V_, *E*_F_, *E*_F-Si_ are conduction band, valance band, Femi energy level of MoS_2_ and p^+^-Si respectively, *e* and *h* denote an electron and a hole.

When a thinner h-BN of 1.6 nm was used, the photon-controlled diode worked as a photodetector. The mechanism differs from acting as a photomemory in the following respect: after removal of light and *V*_G_, the excited electrons can move back from the MoS_2_ to the defect energy levels of h-BN to recombine with the holes there because of the relatively thin tunneling barrier (Supplementary Fig. 6f). The photogating effect of the h-BN disappears. Therefore, the photon-controlled diode changes to the fully-off state and works as a photodetector. To further reduce the response time of this device, a thinner h-BN photogating layer is needed.


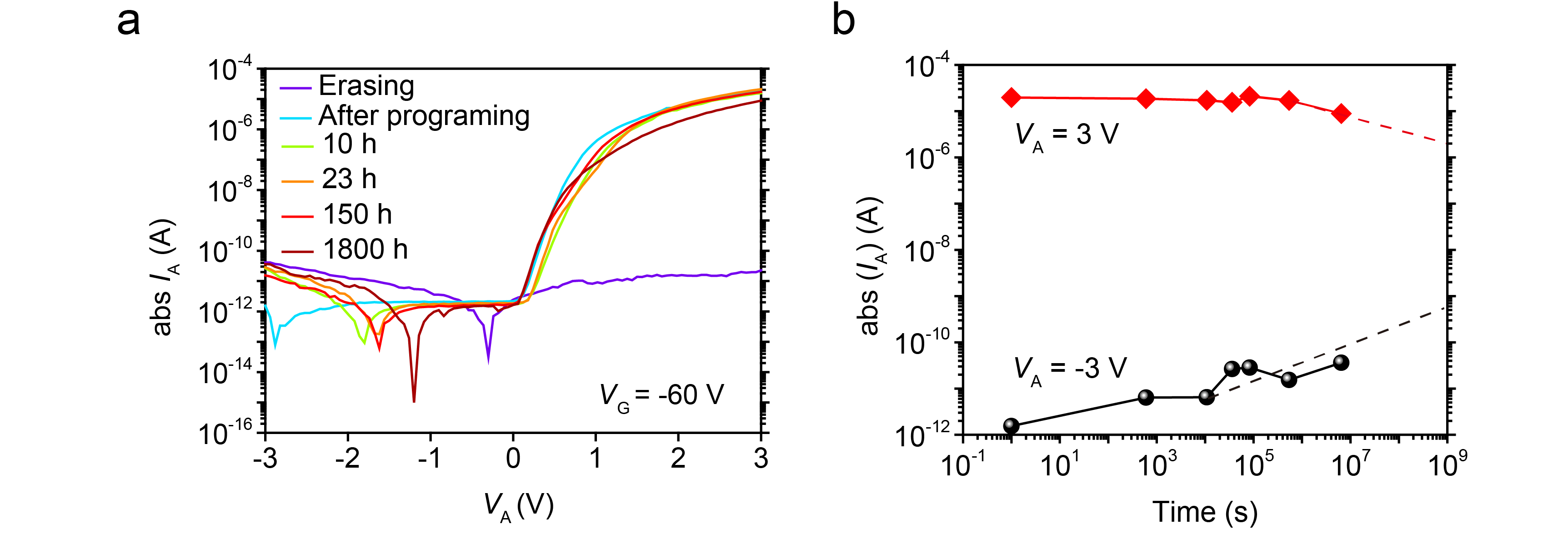


**Supplementary Fig. 7** Retention characteristics. (a) abs (*I*_A_)-*V*_A_ characteristics of the device at *V*_G_ = −60 V after programming. A retention time of more than 1800 hours (6.5 ×10^6^ s) is shown. (b) Extrapolation of the charge retention of the device.

**
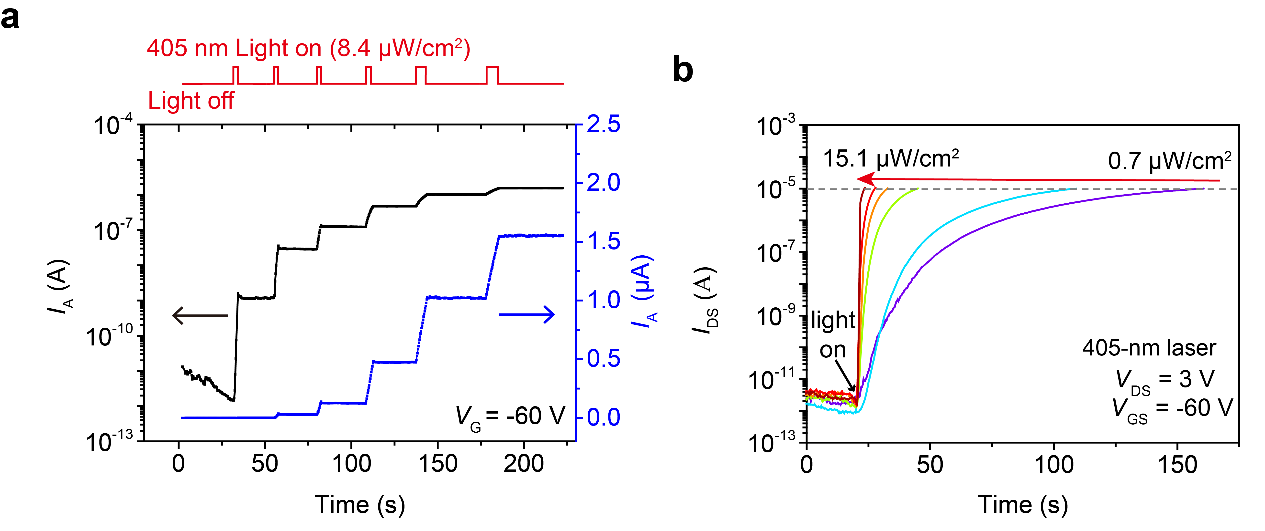
**

**Supplementary Fig. 8** Dynamic behavior of the photomemory under 405 nm light pulses at *V*_A_ = 3 V. After each programming process, the storage current is stable, showing multi-level storage states


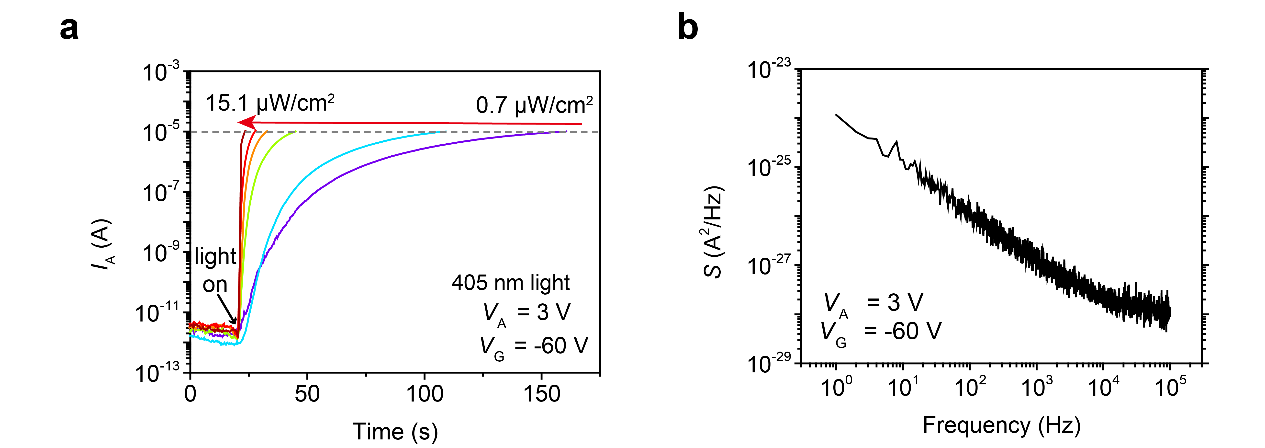


**Supplementary Fig. 9** Photo response and noise measurement of the device. (a) For 405 nm light, the device can detect a power as weak as 0.7 μW/cm^2^. As the power increases, the switching speed increases. When the light is off, the current hardly changes. A storage current (*I*_Store_) of 10^-5^ A is used to calculate the nonvolatile responsivity (*NR*). (b) Noise power spectral density (*S*) of the device at *V*_G_= −60 V and *V*_A_ = 3 V in the dark. A *S* value of 1.1×10^-24^ A^2^/Hz at *f* = 1 Hz was used to calculate the detectivity (*D*^*^).

**
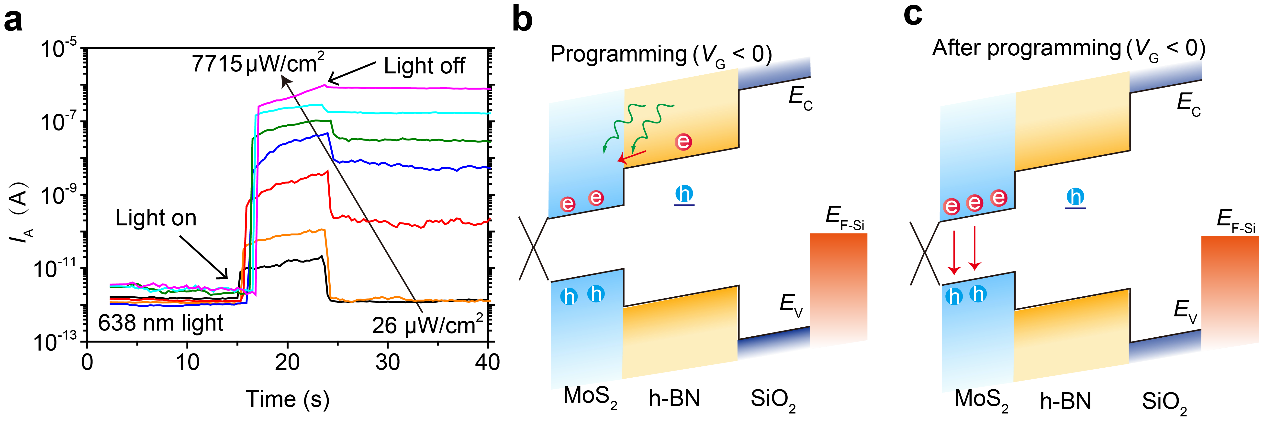
**

**Supplementary Fig. 10** (a) Photo response and mechanism of the device under 638 nm light at *V*_A_ = 3 V and *V*_G_ = –60 V. When the light is off, the current drops. From bottom to top, the power density of the incident light is 26, 97, 339, 778, 1695, 4273 and 7715 μW/cm^2^. (b) and (c) Illustration of the mechanisms of the photomemory under a 638 nm light. Energy band diagrams for the cases of programming and light off. *E*_C_, *E*_V_, *E*_F_, *E*_F-Si_ are conduction band, valance band, Femi energy level of MoS_2_ and p^+^-Si respectively, *e* and *h* denote an electron and a hole.


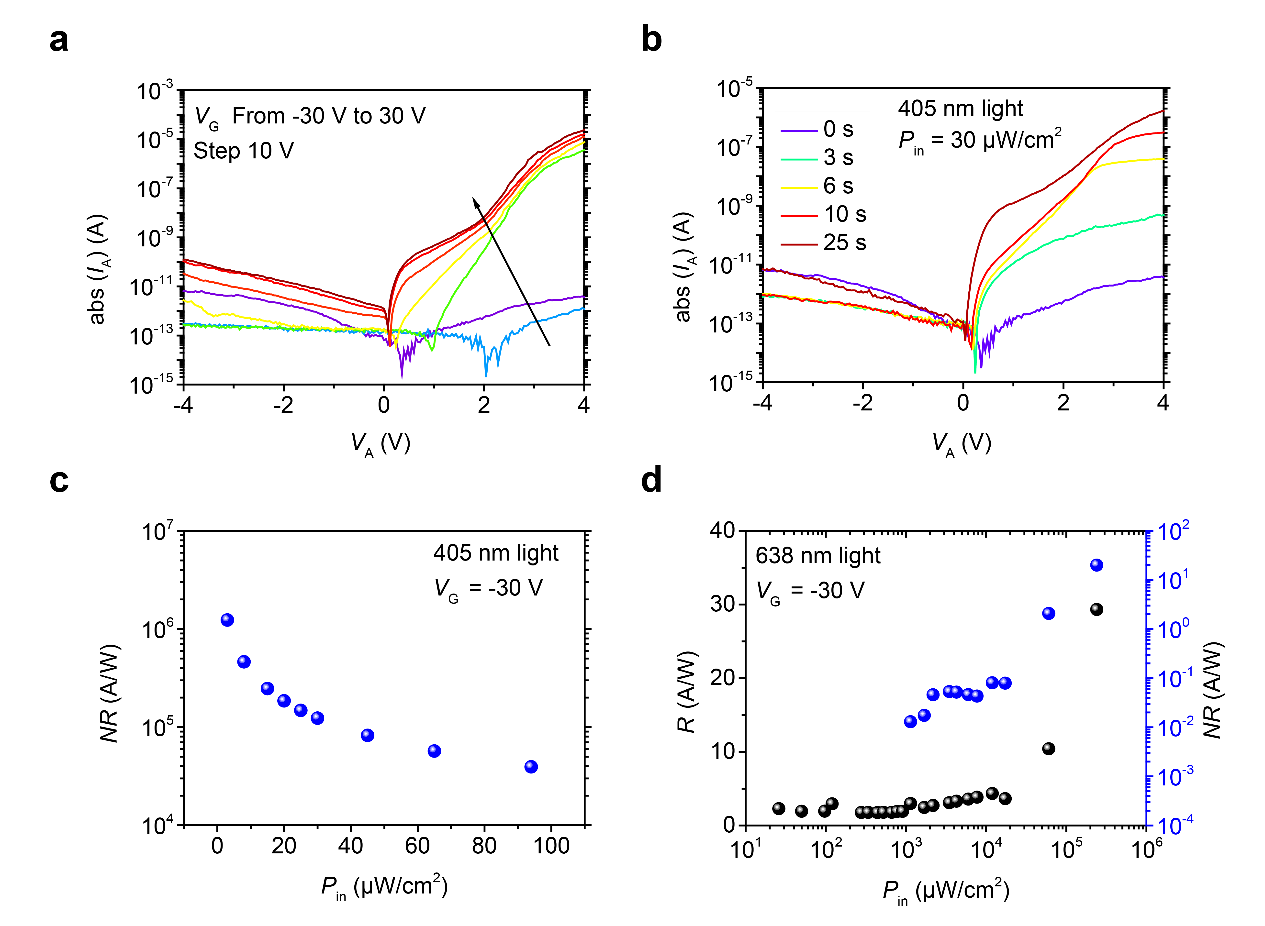


**Supplementary Fig. 11** Characteristics of a photomemory based on a WS_2_ photon-controlled diode. (a) abs (*I*_A_)-*V*_A_ characteristics in the dark with *V*_G_ changing from −30 V to 30 V. (b) abs (*I*_A_)-*V*_A_ characteristics under 405 nm light of 30 μW/cm^2^ at *V*_G_ = −30 V. (c) Nonvolatile responsivity (*NR*) as a function of power density (*P*_in_) under 405 nm light at *V*_A_ = 3 V and *V*_G_ = –30 V. (d) Responsivity (*R*) and *NR* as a function of *P*_in_ under 638 nm light at *V*_A_ = 3 V and *V*_G_ = –30 V.


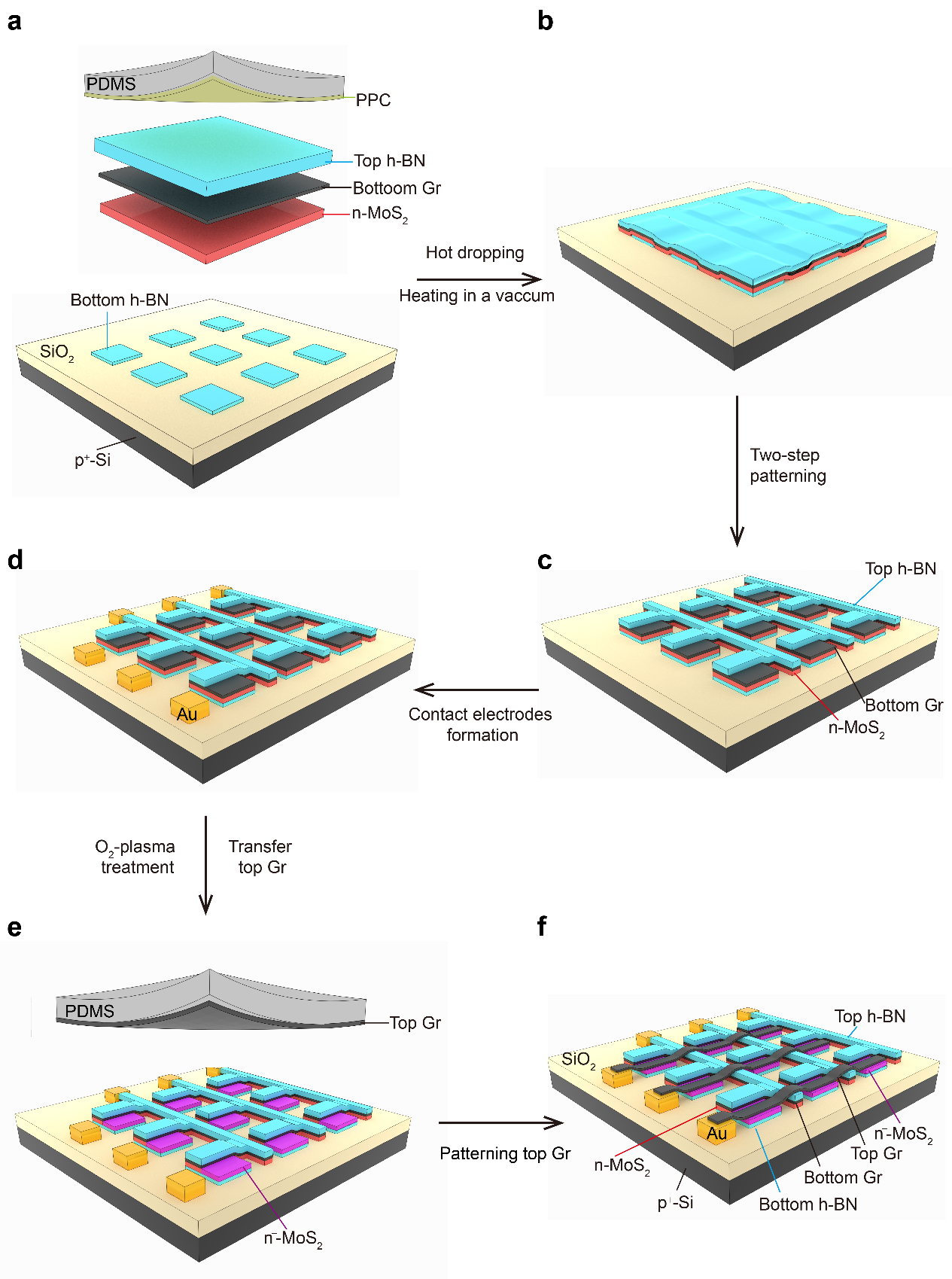


**Supplementary Fig. 12** Illustration of the array fabrication process. (a) h-BN etching mask, bottom graphene and MoS_2_ layers were picked up by a PDMS/PPC layer and stacked on the patterned photogating layer at 130℃. (b) The PPC was removed by heating in a vacuum at 350℃ for 120 min**.** (c) After two-step patterning, the heterostructure was divided into three parts and a p-doped window was formed by EBL, RIE and lift-off processes. (d) Metal contacts (Ti/Au: 5/50 nm) were formed by EBL, RIE, electron-beam evaporation and lift-off processes. (e) p-type doping of MoS_2_ by O_2_ plasma treatment and transferring the top graphene electrode. (f) Pattern of the top graphene electrode.


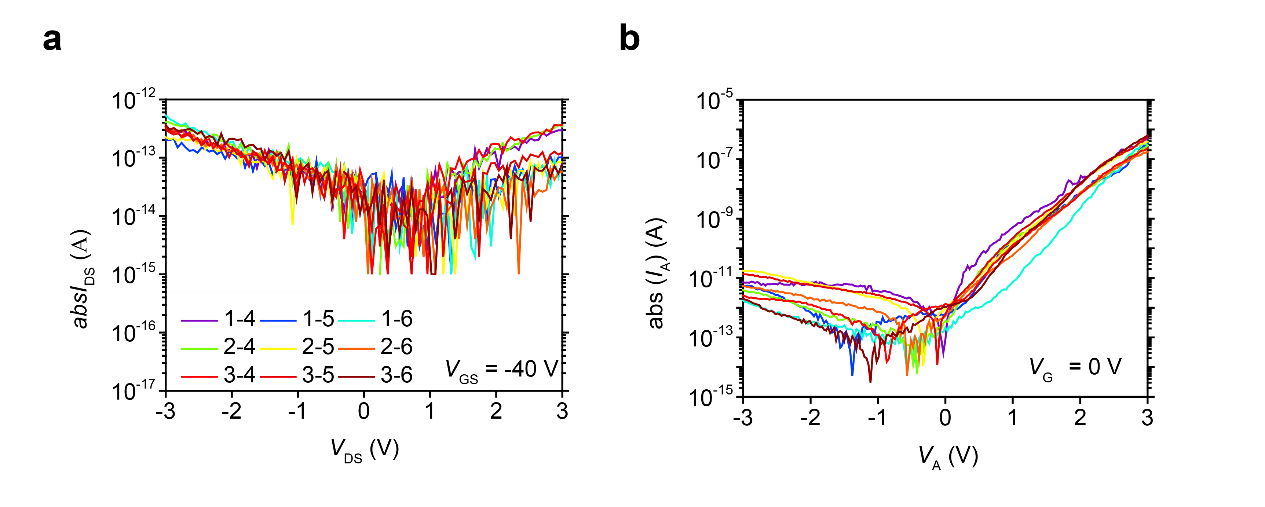


**Supplementary Fig. 13** abs (*I*_A_)-*V*_A_ characteristics of all 9 devices of the array shown in Fig. 5c, in the dark at *V*_G_ = 0 showing a good uniformity.


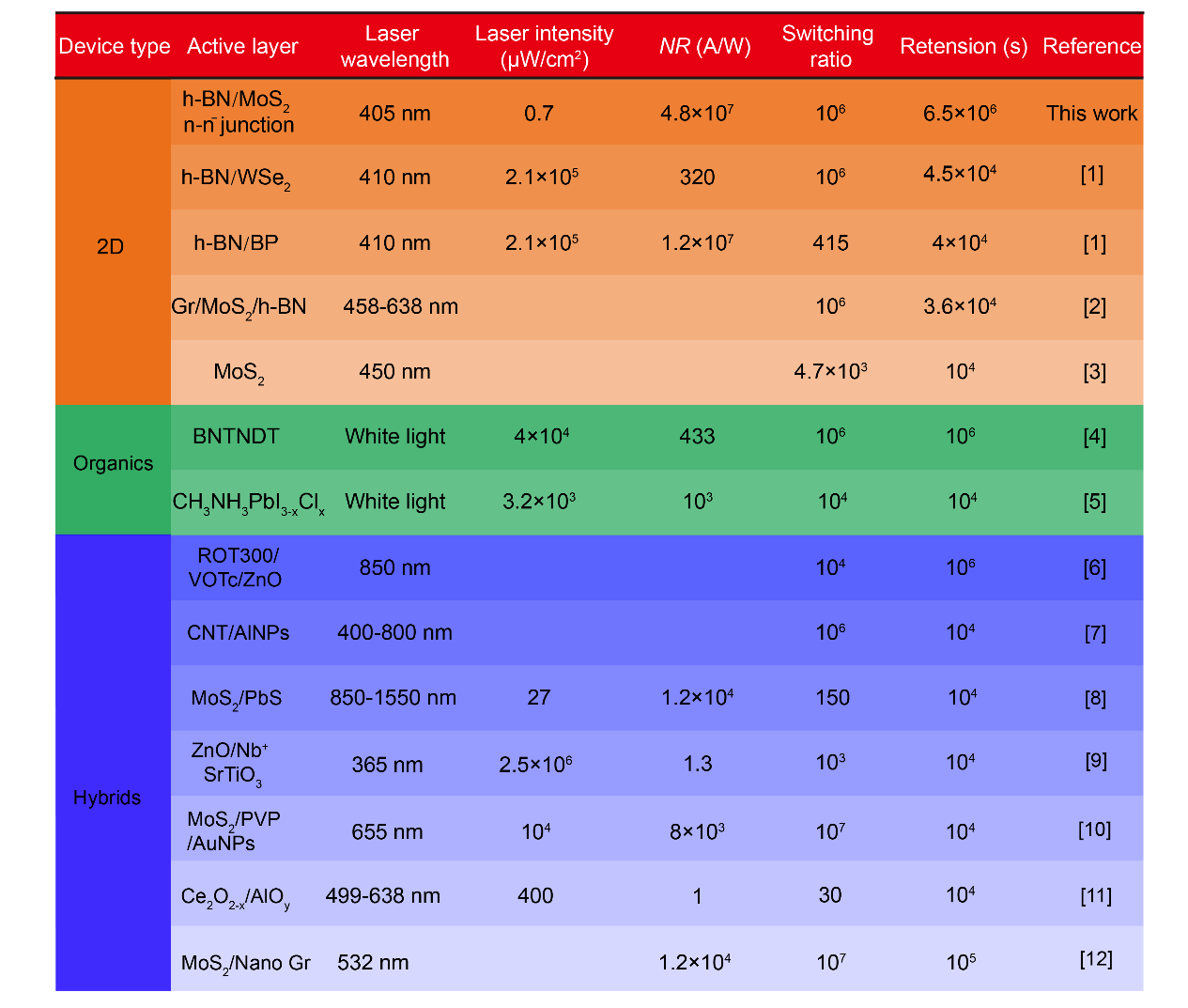


**Supplementary Table. 1** Performance summary of reported photomemory devices. Gr: graphene.

**Reference**

1. Du X, Tao L and Xu J *et al*. Two-dimensional multibit optoelectronic memory with broadband spectrum distinction. *Nat Commun* 2018; **9**: 2966.
2. Tran MD, Kim H and Kim JS *et al*. Two-terminal multibit optical memory via van der Waals heterostructure. *Adv Mater* 2019; **31**: 1807075.
3. Lee J, Pak S and Lee YW *et al*. Monolayer optical memory cells based on artificial trap-mediated charge storage and release. *Nat Commun* 2017; **8**: 14734.
4. Pei K, Ren X and Zhou Z *et al*. A high-performance optical memory array based on inhomogeneity of organic semiconductors. *Adv Mater* 2018; **30**: 1706647.
5. Zhou F, Liu Y and Shen X *et al*. Low-voltage, optoelectronic CH_3_NH_3_PbI_3−_*_x_*Cl*_x_* memory with integrated sensing and logic operations. *Adv Funct Mater* 2018; **28**: 1800080.
6. Wang H, Liu H and Zhao Q *et al*. A Retina-like dual band organic photosensor array for filter-free near-infrared-to-memory operations. *Adv Mater* 2017; **29**: 1701772.
7. Qu T, Sun Y and Chen M *et al*. A flexible carbon nanotube sen-memory device. *Adv Mater* 2020; **32**: 1907288.
8. Wang Q, Wen Y and Cai K *et al*. Nonvolatile infrared memory in MoS_2_/PbS van der Waals heterostructures. *Sci Adv* 2018; **4**: eaap7916.
9. Bera A, Peng H and Lourembam J *et al*. A versatile light-switchable nanorod memory: Wurtzite ZnO on perovskite SrTiO3. *Adv Funct Mater* 2013; **23**: 4977-84.
10. Lee D, Hwang E and Lee Y *et al*. Multibit MoS_2_ photoelectronic memory with ultrahigh sensitivity. *Adv Mater* 2016; **28**: 9196-202.
11. Tan H, Liu G and Zhu X *et al*. An optoelectronic resistive switching memory with integrated demodulating and arithmetic functions. *Adv Mater* 2015; **27**: 2797-803.
12. Zhao J, Wei Z and Yang X *et al*. Mechanoplastic tribotronic two-dimensional multibit nonvolatile optoelectronic memory. *Nano Energy* 2021; **82**: 105692.
